# Mixed Hepatocellular–Neuroendocrine Carcinoma: A Case Report and Literature Review

**DOI:** 10.34172/aim.2023.104

**Published:** 2023-12-01

**Authors:** wala Ben Kridis, ahmed jribi, Rim Kallel, Tahia Boudawara, Afef Khanfir

**Affiliations:** ^1^Department of Medical Oncology, Habib Bourguiba Hospital, University of Sfax, Sfax, Tunisia; ^2^Department of Pathology, Habib Bourguiba Hospital, University of Sfax, Sfax, Tunisia

**Keywords:** Diagnosis, Mixed hepatocellular-neuroendocrine carcinoma, Treatment

## Abstract

Mixed hepatocellular-neuroendocrine carcinoma (HCC-NEC) is a rare entity with a poor prognosis. We report a case of a 44-yearold Tunisian man who was admitted for diffuse abdominal pain. Body computed tomography showed multinodular hepatomegaly. Pathologic findings concluded to HCC-NEC. Clinicians should be aware about this entity. Further collection of case reports is needed to standardize the optimal treatment.

## Introduction

Hepatocellular carcinoma (HCC) is the primary malignant epithelial neoplasm of the liver.^1^ It is the most frequent liver cancer and the seventh most commonly diagnosed malignancy.^1^ HCCs are composed of cells with hepatic differentiation. They often grow in a mosaic pattern in which various cell types are arranged in different architectural patterns. Sometimes they contain additional cells, such as neuroendocrine carcinoma (NEC).^1^ Identification of these components within the HCC is of importance as they provide significant prognostic information. Mixed HCC-NEC are exceedingly rare. They were recently included in the WHO 2019 classification of digestive system tumors within a rare entity of mixed neuroendocrine-non neuroendocrine neoplasm (MiNEN).^2^ HCC-NEC represent 0.40% of primary hepatic cancers.^3^ Here, we present a new case of mixed HCC-NEC with a review of the literature. The aim of this work was to help clinicians to understand its pathology, diagnosis and prognosis in order to provide adequate treatment.

## Case Report

**Figure 1:**
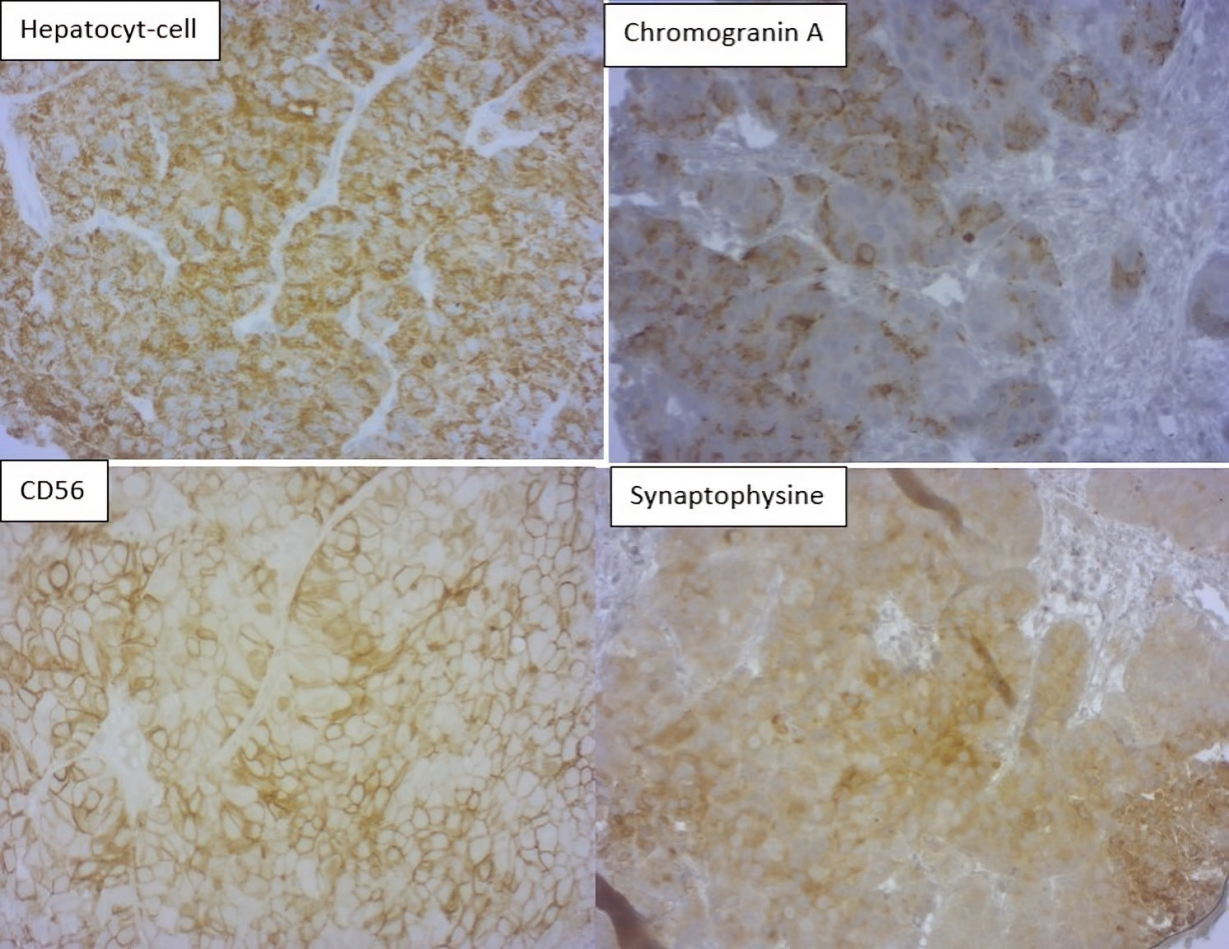
Positive immunostaining for Hepatocyte-cell, Chromogranin A, CD56 and Synaptophysin ( × 200). The tumor was diagnosed as hepatocellular carcinoma with neuroendocrine differentiation with lung and bone metastases. The patient was prescribed sorafenib 400 mg twice a day but he died before starting treatment one month after diagnosis

A 44-year-old Tunisian man with no previous medical, family, and psychosocial history, was admitted for progressively worsening diffuse abdominal pain over 20 days in a context of weight loss. The patient was a heavy smoker (50 pack-years) but not a drinker. His physical examination showed diffuse abdominal tenderness without any disturbance of the bowel motility and without signs of nausea and vomiting. Laboratory examinations including complete blood count (CBC), transaminases (SGOT, SGPT) and lipase were normal. Abdominal ultrasound showed heterogeneous diffuse multinodular hepatomegaly with nodules ranging from 8 to 84 mm. Body computed tomography showed a 3-cm lung opacity in the right apex with a small nodule at the base of the left lung with multinodular hepatomegaly associated with intra-abdominal effusion. Bone scan showed intense uptake in the superior extremity of the right femur. Alpha-fetoprotein level was 8.92 ng/mL (reference range < 7). Hepatitis serology B and C were negative. The differential diagnoses were cholangiocarcinoma or liver metastases from lung cancer. The patient underwent a CT-guided biopsy for further evaluation and diagnosis. Pathologic findings showed tumoral proliferation with focal necrosis constituted of mass cells separated by few abundant and well-vascularized fibrous stroma; tumor cells were large with imprecise limits and well-abundant cytoplasm and voluminous nuclei. Cytonuclear atypia was obvious and mitoses were numerous. Immunohistochemical studies showed intense staining of the tumor cells for hepatocyte cell anti-bodies (anti-Hep-Par1) and CD56, focally staining for chromogranin A and synaptophysin. Cytokeratin-7 (CK7) was positive in few cells and immunostaining was negative for cytokeratin-20 (CK20), TTF1 and P63. Interestingly, the tumor cells were stained with the hepatocyte cell and the neuroendocrine markers at the same time (Figure 1).

## Discussion

After an extensive literature search, from January 1970 to September 2021, we identified 35 cases of mixed HCC-NEC. As far as we know, our case seems to be the 36th case of mixed HCC-NEC reported in the literature and the fourth reported case of mixed HCC-NEC combined type in its intermediate subtype.^4^ After an exhaustive review of the 35 cases found in the literature and our case, we were able to draw some interesting conclusions. In fact, 61% of cases were reported from Asia. Men were more likely to present with this rare pathology with 89% (32/36) compared to women with 11% (4/36).^4^ The median age of manifestation of this disease was 61 years (range: 19 to 84). Approximately 75% (27/36) of the reported cases had hepatitis (HCV in 56% and HBV in 37%). The precise diagnosis was based predominantly on postoperative pathological examination findings. The majority of the reported cases were preoperatively diagnosed as HCC and re-diagnosed as combined or collision HCC-NEC tumors after resection; 28 cases (72%) were diagnosed as mixed tumors based on the pathologic findings after surgery, 4 cases (11%) on the pathologic data of core biopsy, 2 cases (6%) on the pathologic data of autopsy and one case on the pathologic data of fine needle aspiration.^5^ This demonstrates the difficulty of obtaining a correct preoperative diagnosis for these tumors. Interestingly, our case diagnosis was based on the data of simple biopsy. The diagnosis of HCC-NEC is retained in case of cells staining of the hepatocyte cell and the neuroendocrine markers (chromogranin, synaptophysin, CD56) at the same time.^5^

The mean size of the tumor was approximately 6.7 cm. The majority of mixed HCC-NEC tumors presented as a combined type in 72% of cases (26/36) followed by collision type in 25% of cases (9/36). Only one case was a mixture of both combined and collision types.^5^

From the published cases of combined type, only 3 cases and ours were found to be in an intermediate subtype (Table 1).^4,5^ Elevation of serum AFP was reported in 61% of cases (22/36).

**Table 1:** Reported Cases of Combined Types of Mixed Hepatocellular Carcinoma-Neuroendocrine Carcinoma in their Intermediate Subtype

**Study/** **Year**	**Age**	**Diagnosis**	**Size (cm)**	**Tumor Markers**	**Treatment Received**	**Metastasis/Recurrence**	**Survival** **(months)**	**Region**
Nomura et al^3^ 2016	58	Resection	4.3	AFP ↑	Surgical resection	NR	19.6	Asia
	63	Resection	3	AFP ↑	Surgical resection	NR	24	Asia
Lan et al^5^ 2021	39	Resection	21	AFP ↑	TACE then surgical resection then Etoposide and cisplatin-based CT.	NR	6	Asia
Our case 2021	44	Biopsy	Multifocal (biggest = 7.4)	AFP slightly↑	-	Metastatic lung + bone (right femur extremity) at diagnosis	1	North Africa (Tunisia)

NR, not reported; TACE, trans-arterial chemoembolization; (↑), elevated.

 Because of its rarity, there is no consensus about the management of this entity. Surgical resection provides long-term survival. However, local or distant relapse is common after surgical resection and is usually fatal.

In our case, due to lack of special data about the management of this rare entity, treatment consisted of management of inoperable HCC and the patient was prescribed palliative targeted therapy in the form of sorafenib. Our patient died one month after diagnosis before starting sorafenib. The 1-year survival rate of the patients was only 25% in our literature review. The mean survival was 9.3 months with the worst reported survival equal to 7 days and the best reported survival equal to 33 months.^5^

## Conclusion

 Mixed HCC-NEC is an ambiguous entity. According to our knowledge, our case seems to be the 36th case of mixed HCC-NEC reported in the literature and the 4th reported case of mixed HCC-NEC combined type in its intermediate subtype. It is uncertain how these neoplasms develop and how they should be treated. Further collection of case reports is needed to standardize the optimal treatment.
